# Seeking connection: a mixed methods study of mental well-being and community volunteerism among international migrants in Japan

**DOI:** 10.1186/s12889-020-09381-2

**Published:** 2020-08-20

**Authors:** Russell Miller, Ken Ing Cherng Ong, Suhyoon Choi, Akira Shibanuma, Masamine Jimba

**Affiliations:** Department of Community and Global Health, Graduate School of Medicine, The University of Tokyo, 7-3-1 Hongo, Bunkyo-ku, Tokyo, 113-0033 Japan

**Keywords:** International migration, Mental health promotion, Well-being, Mixed methods, Japan

## Abstract

**Background:**

International migration is a stressful process for which non-Western nations are a growing destination. However, little attention has been paid to the psychological well-being of international residents or their local integration in such settings. Prosocial behavior, like volunteering in one’s local community, has been demonstrated to improve mental health in native populations. Volunteerism may be a mental health promotion strategy applicable to non-native migrants as well. In order to assess such a hypothesis, this study investigated the mental well-being of international migrants living in Tokyo, Japan, who did or did not participate in formal volunteering.

**Methods:**

This convergent mixed-methods study assessed mental well-being with the Warwick-Edinburgh Mental Well-being Scale (WEMWBS) and qualitative follow-up interviews. Migrants who contributed their time to structured volunteering roles (formal volunteers, *n* = 150) were recruited from local non-profit organizations. Migrants who did not formally volunteer (*n* = 150) were recruited from social media sites. In parallel, a nested participant sample from both groups (*n* = 20) were interviewed about their satisfaction with life in Tokyo.

**Results:**

After adjusting for sociodemographic characteristics, volunteering was not associated with higher mental well-being score (*p* = 0.215), but instead, not feeling isolated (*p* = 0.008), feeling connected to Japan (*p* = 0.001) and employment satisfaction (*p* < 0.001) were significantly associated with mental well-being. Follow-up interviews similarly demonstrated that migrants participated in various social activities to promote personal well-being and deeper social connections with Japanese, regardless of volunteering status.

**Conclusions:**

Volunteering status itself was not significantly associated with mental well-being score among international migrants in Japan after adjusting for potential confounding variables. Beyond volunteering, having deeper social connections with the Japanese community is a key to promoting migrant mental well-being.

## Background

Humans have never had more mobile life trajectories. International migrants constitute a vulnerable global minority and, in 2019, 3.5% of the world’s population [1]. The push and pull factors that characterize migration are stressful as much as they are linked with the pursuit of opportunity and a ‘good life’ abroad [2, 3]. Evidence from global health has demonstrated that migrant mental health suffers most due to restrictive entry and integration policies [4]. Taking this stress burden into consideration, migrants’ right to mental health, along with recommendations about their integration into host societies, were most recently enshrined in the 2016 *Global Compact for Safe, Orderly and Regular Migration* by the United Nations General Assembly [5]. While recent research initiatives have conceptualized international migration as a social determinant of physical and mental health [6], emerging destinations of migration have been slow to embrace such a framework.

Facing a severe demographic contraction, Japan, as the world’s third-largest economy, is in the midst of a migration transition as part of globalization. The migrant population increased to 2,731,093 foreign residents or 2.2% of the Japanese population in 2018 in which three largest groups by nationality were Chinese (28.0%), Korean (16.5%) and Vietnamese (12.1%) [7]. However, the psychological settlement of migrants in Japan has been left mostly unaddressed by ad hoc migration policy and an inadequate research base [8, 9]. A recent systematic review regarding the positive mental health of resident migrants in Japan [10] found the existence of, or lack thereof, social support networks to be the most common association with mental well-being [11]. Furthermore, the Migration Policy Index which tracks the favorability of receiving countries based on eight parameters (including health and labor market mobility), ranks Japan as one of the least favorable migration destinations in the Organization for Economic Co-operation and Development (OECD) [12]. This poor standing is contrary to the attention that the national government has placed on recruitment and retainment of specialized blue-, pink- and white-collar foreign workers [13].

The apparent discrepancy between migration policy goals and suboptimal mental health outcomes might be addressed effectively at the community level. For example, prosocial civic engagement activities such as volunteering have been demonstrated to be closely associated with improved mental well-being [14, 15]. Although the causal mechanisms of this association remains debated, positive psychosocial outcomes such as finding meaning through social relationships, are thought to play a role [16]. While it is conceivable that higher mental well-being exists among volunteering migrants, such investigations have rarely been done, especially outside of Western migration settings [17]. The factors associated with positive mental health in this minority are likely complex and quantitative data alone may not provide proper context. Therefore, a mixed methods design was employed to quantitatively measure the mental well-being of migrants in Tokyo, Japan and, at the same time, qualitatively explore the perceptions of satisfaction with life in the city that simultaneously informed findings on participation and mental well-being.

## Methods

**Fig. 1:**
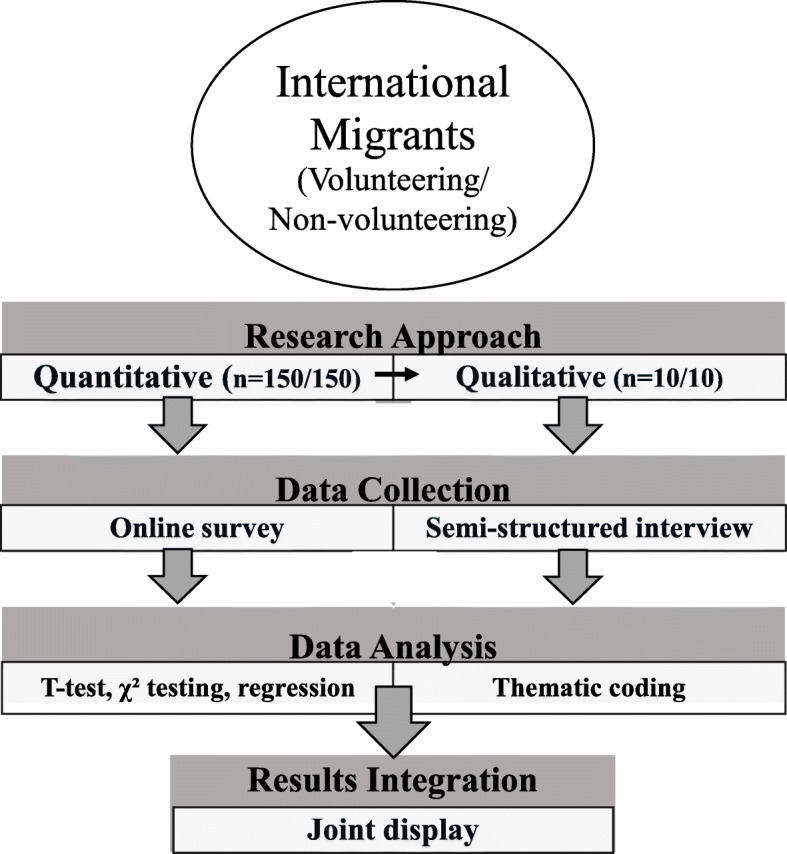
Flowchart of recruitment, data collection, analysis and integration

This study employed a convergent mixed methods study design to investigate the mental well-being of international migrants who did and did not formally volunteer in Tokyo, Japan. Quantitative and qualitative approaches were employed with concurrent data collection, independent data analysis and final integration. Furthermore, a community-based participatory research (CBPR) sub-framework was also utilized to co-create knowledge with community stakeholders [18]. This holistic research framework is outlined in Fig. 1.

The study population was international migrants living and/or participating in formal organizations within Tokyo Metropolis between June and September 2019. Formal volunteering was adapted from Jenkinson et al. and was defined as an activity undertaken to benefit the community with organizations that have structured volunteer positions [16]. Inclusion criteria were having lived in Japan for more than 3 months (minimum stay requirement for non-tourist visas), having non-Japanese citizenship, and being 20 years old or over. The sample size calculation for this study was based on investigation with Warwick-Edinburgh Mental Well-being Scale (WEMWBS) of Indian international migrants in the United States who did and did not participate in local ethnic group organizations [19]. Sample size was calculated using the mean difference of WEMWBS scores between equal-sized groups with a statistical power of 80% and a significance level set at 5%. As this was an online survey, a drop-out rate of 20% was factored into the calculation yielding a sample size of 300 migrants made up of two groups by volunteering status (*n* = 150 each).

Working in partnership with two accredited non-profit organizations (NPOs), volunteering migrants group were recruited from organizations at that offered volunteering opportunities in English. These community organizations were: TELL Japan, an NPO offering mental health counseling and outreach in English; and Second Harvest Japan, a non-governmental food bank operating in English and Japanese. Both organizations are headquartered in central Tokyo. Non-volunteering migrants were self-identified online via convenience sampling with postings on the Facebook page of each NPO, Tokyo expatriate Facebook groups, as well as on LINE and Weibo messaging application networks.

An online survey-sharing platform, Google Forms, was used to create a questionnaire consisting of 52 items with a landing page consent form and a screening question to confirm either residence or participation in formal groups within Tokyo. The first 12 items of the questionnaire were sociodemographic, next was the WEMWBS [20] and the remaining items had been adapted from specific subscales of the Immigration Policy Lab Integration Index (IPLII) [21]. Official permission to use the WEMWBS was obtained from the scale’s administrator.

The WEMWBS assesses mental well-being and contains 14 positively worded items with a 5-point response scale ranging from “none of the time” to “all of the time”. It assesses positive and negative affect with a total possible score of 70 and has been previously validated in two migrant populations residing in the UK [22]. The reliability of the WEMWBS in the current study was high with a Cronbach’s alpha of 0.92. The ratio of volunteering to non-volunteering migrants was monitored during data collection to dictate targeted recruitment to maintain roughly balanced study groups.

Questionnaire data were analyzed using t-testing to identify statistically different variable outcomes between groups as well as multiple linear regression modeling to identify factors associated with mental well-being. The regression model of mental well-being was created based on theoretical concepts gleaned from a review of the literature after controlling for important sociodemographic variables [11]. All analyses were performed with Stata v13.1 (College Station, TX: Stata Corp LP). This study followed the Strengthening the Reporting of Observational studies in Epidemiology (STROBE) [23] for cross-sectional assessment.

At the end of the online questionnaire, migrants were asked to enter their email if interested in a follow-up interview about their satisfaction with life in Tokyo (which carried an incentive of a 1000 yen gift card). A nested sample size of twenty interviews was estimated to provide enough new information to reach data saturation for robust analysis based on guidelines for the qualitative study of migrants [24]. The sociodemographic information of migrants who submitted email addresses were screened first (to ensure close to 50% participated in formal volunteering) by volunteer status, nationality, sex and age for maximum variation sampling via email. A single interviewer (RM; male) adjusted the interview guide based on pilot testing results and later led and voice-recorded semi-structured interviews (either online or in-person) with migrants after receiving informed consent. Each migrant was assigned an ID number to maintain their confidentiality. Interviews lasted an average of 45 min.

For qualitative interviews, verbatim transcription of each interview was assembled with a standardized format and then validated by member-checking with the interviewee. Digital and paper transcripts as well as voice-recordings were kept strictly confidential in a desk to which only the primary researcher (RM) had access. Final transcripts were coded parallel with another researcher (SC) as part of reflexive thematic analysis seeking to identify common trends in reasoning across transcripts [25]. Peer validation with a third researcher (KICO) was used to finalize codes. This study is in accordance with the Consolidated Criteria for Reporting Qualitative Research (COREQ) [26].

Additionally, one author (RM) completed participant observation by regularly volunteering with both NPOs for 5 months around the data collection period, and was not separated nor identified as a researcher by NPO staff. Over 100 volunteer-hours were logged with field notes on conversations with volunteers about their motivations for their social participation. The Research Ethics Committee of the Graduate School of Medicine, the University of Tokyo approved this study (serial number 2019066NI). All NPOs and migrants who either participated in the survey or interviews provided electronic or written consent.

### Integration

Final results from both independently analyzed methods were integrated with equal weight to compare and contrast meta-inferences [27]. Integration findings informing the same constructs were presented together as joint-displays. Quantitative and qualitative observations were presented next to each other with a meta-inference below [28]. Guidelines included in the Mixed Methods Appraisal Tool (MMAT) [29] were followed.

## Results

In total, 300 survey responses were collected. After data cleaning, eight entries were found to be missing substantial data and were excluded from further analysis leaving a total of 292 valid responses. The sociodemographic characteristics of the quantitative and qualitative participants are described in Tables 1 and 2, respectively.

**Table 1:** Sociodemographic characteristics of international migrants; questionnaire (*n* = 292)

	Non-volunteers (***n*** = 145)		Volunteers (***n*** = 147)		
**Mental well-being score**, mean [SD]	47.0 [10.5]		50.3 [8.7]		**
**Age**, mean [SD]	33.5 [10.9]		35.8 [13.1]		
	**n**	**%**	**n**	**%**	
**Sex**					
Male	59	(40.6)	60	(40.8)	
Female	86	(59.4)	87	(59.2)	
**County of citizenship**					
Western	82	(57.3)	89	(61.1)	
Asia	53	(37.1)	45	(30.2)	
Other	8	(5.6)	13	(8.7)	
**Education level**					
High School or less	8	(5.5)	7	(4.8)	
University/College	67	(46.2)	81	(55.1)	
Post-Graduate	70	(48.3)	59	(40.1)	
**Living Situation**					*
Living alone	70	(48.3)	57	(38.8)	
Living with relatives	43	(29.7)	61	(41.5)	
Living with others	32	(22.1)	29	(19.7)	
**Martial Status**					*
Not Married	88	(60.7)	75	(51.0)	
Married with non-Japanese spouse	35	(24.1)	35	(23.8)	
Married with Japanese spouse	22	(15.2)	37	(25.2)	
**Have Children**					*
No	117	(80.7)	103	(70.1)	
Yes	28	(19.3)	44	(29.9)	
**Migration motivation**					
Study	63	(43.4)	59	(40.1)	
New job	53	(36.6)	51	(34.7)	
Other	29	(20.0)	37	(25.2)	
**Monthly Household Income** (Japanese yen)					*
< 250 K	52	(40.9)	40	(27.2)	
250-550 K	44	(30.3)	52	(35.4)	
≥ 550 K	31	(21.4)	39	(26.5)	
No response	18	(12.4)	16	(10.9)	
**Settlement in Japan** (years)					**
≤ 5 years	92	(63.4)	74	(50.3)	
> 5 years	53	(36.6)	73	(49.7)	
**Future Settlement in Japan** (years)					
≤ 5 years	67	(46.2)	61	(41.5)	
> 5 years	28	(19.3)	37	(25.2)	
Undecided	50	(34.5)	49	(33.3)	
**Employment**					
Student	39	(26.9)	42	(28.6)	
Full-time work	73	(50.3)	71	(48.3)	
Other	33	(22.8)	34	(23.1)	
**Feeling isolated in Japan**					*
No	59	(40.7)	44	(29.9)	
Yes	86	(59.3)	103	(70.1)	
**Employment Satisfaction**					**
Neither satisfied nor dissatisfied	65	(44.8)	44	(29.9)	
Somewhat satisfied	50	(34.5)	64	(43.5)	
Very satisfied	30	(20.7)	39	(26.5)	
**Feeling connected to Japan**					
No	38	(26.2)	19	(12.9)	***
Yes	107	(73.8)	128	(87.1)	

* *p* < 0.05; ** *p* < 0.01; *** *p* < 0.001

**Table 2:** Sociodemographic characteristics of international migrants; interviews (*n* = 20)

	Non-volunteers (***n*** = 9)		Volunteers (***n*** = 11)	
**Age**, mean [SD]	34.0 [9.9]		36.8 [15.1]	
	**n**	**%**	**n**	**%**
**Sex**				
Male	3	(33.3)	3	(27.3)
Female	6	(66.7)	8	(72.7)
**County of citizenship**				
Western	1	(11.1)	4	(36.4)
Asia	5	(55.6)	4	(36.4)
Other	3	(33.3)	3	(27.3)

### Quantitative

Of the 292 valid responses, 147 were purposively sampled volunteering migrants and 145 did not participate in formal volunteering (Table 1). Similar trends were found for all sociodemographic data among both groups. The average group ages were not statistically different at roughly 35 years old for both groups. Ages were concentrated between 20 and 39, accounting for about 70% of the migrants for both groups. Overall, more women than men participated in the study (59.2% vs 40.8%) and both groups tended to have completed tertiary education (95%), not be married (> 50%), without children (> 70%), came to Japan for study (> 40%) and were now working full-time (> 48%). Fewer non-volunteers had settled in Japan for more than 5 years versus volunteers (36.6% vs 49.7%, *p* < 0.01). More than 75% of both groups were either undecided about their future settlement time in Japan or were planning to leave in 5 years or less.

Responses were more varied in terms of living situation and income. A substantial proportion of non-volunteers lived alone while a large proportion of volunteers lived with relatives (29.7% vs 41.5%, *p* < 0.05). While the income item only had a 79% response rate for both groups, the largest proportion of non-volunteers tended to have a household income below 250 thousand yen (about $2400) per month (40.9%) versus a minority of volunteers (27.2%, *p* < 0.05).

The majority of both groups felt isolated in Japan at least some of the time (59.3% of non-volunteers vs 70.1% of volunteers, *p* < 0.05). Neither group reported dissatisfaction with their employment but more non-volunteers had neutral feelings (44.8% vs 29.9%, *p* < 0.01) and most migrants felt some connection to Japan (73.8% vs 87.1%, *p* < 0.001). The mean WEMWBS score for the entire study population was 48.7 points. When divided by volunteering status, mean scores were 47.0 and 50.3 for non-volunteers and volunteers, respectively (*p* = 0.004). In the multiple linear regression model of mental well-being controlling for important sociodemographic characteristics (Table 3), compared to non-volunteers, volunteering status was not found to be associated with mental well-being score (B = 1.3; *p* = 0.215). Finally, the integration-related items significantly associated with mental well-being score were not feeling isolated (B = 3.2; *p* = 0.008), feeling connected to Japan (B = 4.9; *p* = 0.001) and employment satisfaction (B = 4.9; *p* < 0.001).

**Table 3:** Factors associated with mental well-being score

Adjusted R-squared = 0.2638	B	***p***-value	95% CI
Volunteering status (vs. non-volunteer)			
Volunteer	1.3	0.215	(−0.7, 3.3)
Sex (vs. male)			
female	−0.3	0.784	(−2.3, 1.7)
Age	< 0.1	0.458	(−0.1, 0.1)
Education level (vs. secondary or less)			
University/college	3.4	0.139	(−1.1, 8.0)
Post-graduate	3.3	0.161	(−1.3, 7.9)
Marital status (vs. not married/divorced/widowed)			
Married with non-Japanese spouse	2.2	0.078	(−0.2, 4.7)
Married with Japanese spouse	1.6	0.272	(−1.3, 4.5)
Time settled in Japan (vs. ≤5 years)			
> 5 years	−1.9	0.090	(−4.2, 0.3)
Feeling isolated in Japan (vs. isolated)			
Don’t feel isolated	3.2	**0.008**	(0.8, 5.5)
Feeling connected to Japan (vs. not connected)			
Feel connected	4.9	**0.001**	(2.1, 7.7)
Employment satisfaction (vs. neither satisfied nor dissatisfied)			
Moderately satisfied	4.9	**< 0.001**	(2.7, 7.2)
Very satisfied	9.3	**< 0.001**	(6.7, 12.0)

### Qualitative

Qualitatively, 11 volunteering migrants and 9 non-volunteering migrants participated in face-to-face interviews (Table 2). Three migrants partook in online video interviews due to scheduling restraints; all other interviews were completed face-to-face. The interview setting did not seem to impact interview responses. Both groups tended to be students who had already finished tertiary education and had stayed in Japan for 1 to 3 years or less in both groups. While “over 10 years” was the most common answer about the desired length of stay in Japan for volunteers, ‘undecided’ length of stay was the most common answer for non-volunteers. About half of each group were from Asia and mental well-being scores were higher for volunteers than non-volunteers.

Migrants’ satisfaction with and participation in their local community was explored to address the research question about why migrants do or do not socially participate. Several prominent themes arose from thematic analysis of interview transcripts.

#### Seeking connection

After voicing frustration with language barriers and praise for the politeness of Japanese society, seeking deeper social interactions and connections became the overarching theme of the interviews, regardless of reported Japanese ability.

*“But since I want to live here, I want to get a bit closer, settle down, deeper.” - 12B, female, 57 years old (y.o.), volunteering*

*“Well for my part, maybe I just need to keep trying to reach out. Otherwise I won’t meet anyone here basically.” - 1D, male, 34 y.o., non-volunteering*

Migrants who engaged in prosocial activities, including and excluding formal volunteering, as well as long-term residents of Japan, expressed a similar desire for social links with Japanese people. Migrants reported connections with locals never developing beyond a theme characterized as “*surface interactions”*. Similarly, a concept of *“the neighbor”* was reported as the archetype of social connection that they would like to possess to feel more satisfied with their life. Perceptions about the reasons for a lack of connection ranged from guesses about inflexible cultural norms to English language limitations of Japanese counterparts.

#### Prosocial engagement

Migrants also discussed their motivations for prosocial engagement in Tokyo through the prism of personal well-being.

*“I am giving back but I think the first thing is for myself. … . I like to do things that I'm reasonably good at and make me feel somewhat professional.” - 13N, female, 72 y.o., volunteering*

*“I would love to [do volunteering activities] … But speaking about my spare time … I really love reading, studying foreign language.” - 13N, female, 24 y.o., non-volunteering*

Various volunteering and other activities gave meaning, an indicator of mental well-being, to each migrant as they moved through their lives in Tokyo. For example, joining events put on by an interviewee’s ethnic group, business-interest networking, or hiking in nature were all common activities. By participating in these events, migrants reported meeting like-minded foreign and Japanese individuals who shared common interests, although lasting, deep connections were still lacking.

#### Employment satisfaction

Finally, employment satisfaction was an essential theme that most migrants discussed.

*“And I thought it would be better. But it got worse because I didn't have a job and I was now without a purpose.” - 5I, female, 32 y.o., volunteering*

*“I got a job offer from a company that is, let me just say it’s an oasis... It’s a miracle in Japan.” - 15C, female, 26 y.o., non-volunteering*

A few migrants reported feeling distressed if they were unable to utilize their expertise due to a mismatch in job skills to available opportunities. These migrants were planning to leave Japan in the next few years for career growth abroad. Conversely, if they were content with their employment situation, migrants were even more focused on seeking personal well-being through social interaction in Japan.

### Integration

Three main findings were brought into focus by integrating the results of independent quantitative and qualitative analyses.

**Fig. 2:**
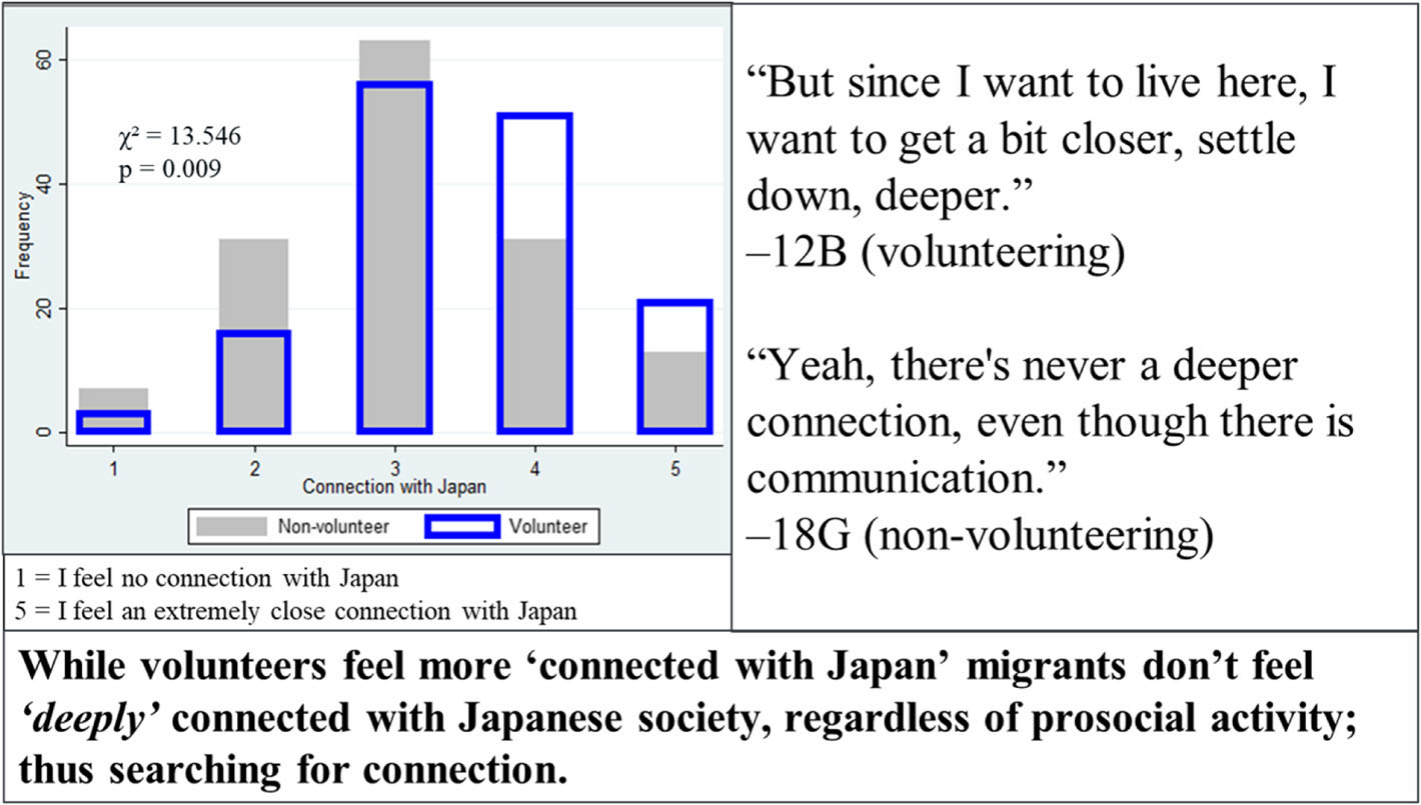
Joint display of volunteering and connection with Japanese society

One notable integrated finding was a divergent one related to the connection to Japan felt among volunteers and non-volunteers (Fig. 2). While volunteering status was not associated with mental well-being score, volunteering status was strongly associated with a connection to Japan (*p* = 0.009, figure left panel). However, almost all of the migrants focused on how lack of social connection was limiting their satisfaction in Japan (right panel). This discordant finding presents the desire of settled migrants to make a *“deeper”* social connection with the domestic majority which exceeds the superficial concept of the connection with the host nation as only a place of residence.

Merging of the data revealed the complexity of the link between volunteering and mental well-being score (see Additional file 1). The box and whisker plot in the left panel shows the statistically different yet comparable mental well-being scores of volunteering migrants and those that did not. The red lines on the graph add context by indicating thresholds of flourishing (high mental well-being: 58 points) and a score threshold suggestive of probable depression (moderate to poor mental well-being: 41 points) [30]. While qualitative interviews did not directly contradict the incremental mean increase in mental well-being among volunteers, both volunteers and non-volunteers spoke about various self-motivated activities to promote their own well-being. This finding confirmed the a priori expectation that volunteers would have better mental well-being scores than non-volunteers but with a self-first rather than altruistic motivation.

The final joint display (see Additional file 2) explores employment satisfaction among migrants and its association with mental well-being. The bar graph in the figure represents the incremental increases in mental well-being score stratified by volunteering status from “moderately well” to “very well”. In a multiple linear regression model of mental well-being, satisfaction with employment was demonstrated to have the strongest association. Migrants described satisfaction about their job in Japan as a foundation from which to branch out and search for a like-minded community to socially engage with others. Qualitative and quantitative conclusions were aligned for this meta-inference about employment.

## Discussion

International migrants to Japan who volunteered, did not feel isolated, had employment satisfaction and felt a strong connection with Japan were found to enjoy higher mental well-being. However, the association between volunteering and mental well-being score was not significant after adjusting for the latter factors. Prominent themes from both datasets (quantitative and qualitative) often complimented each other but divergence emerged when perceptions related to settlement among international migrants were examined. According to mixed methods integration, challenges remain in maintaining migrant mental well-being in a non-native Japanese setting, mainly via unmet social needs regardless of volunteering status.

The positive mental well-being outcomes related to volunteering among migrants had many parallels with studies of volunteering among non-migrants. According to the World Happiness Report 2019 [31], powered by Gallup polling of over one million participants from 130 countries, those who volunteered were more likely to have higher well-being scores. Similarly, non-migrants who participated in volunteering had higher mental well-being than non-volunteers in a well-powered longitudinal investigation in the US [32]. That study also found that people with better baseline well-being scores dedicated more hours to volunteering over time. The literature suggests that the positive association between well-being and volunteering is a cross-cultural phenomenon. This conclusion is further supported by the positive results observed in the present study sampling migrants with diverse national backgrounds.

Volunteering migrants in the current study reported that volunteering was an activity they enjoyed before living in Japan. This finding extended to volunteering with one’s ethnic group was noted in the current analysis and has been previously detailed in the migration literature [19]. Another longitudinal panel study of older UK volunteers reported their involvement with community groups as an innate motivation [14].

It should be noted with caution that the direction of causality between volunteering and positive mental health outcomes remains debated. Some research has shown that people with better baseline mental well-being are more likely to volunteer [33]. On the other hand, those with depression are most likely to see benefit from dedicating time to volunteering [34]. A few qualitative participants in the present study also mentioned their keen awareness of mental health and the importance of self-care whether that included volunteering or not. Further research into the mechanism the association between volunteering and mental well-being is warranted.

Investigation of factors related to both mental well-being score confirmed the importance of economic integration for migrants. The migrants in this study were likely to be considered skilled workers by the Japanese government due to their previous experience in Japan, domestic studies and/or tertiary-level education [35]. As volunteering migrants reported higher levels of income, this may suggest migrant volunteers were more financially stable than non-volunteers and thus had time to volunteer. However, it is also likely that all migrants who took time to participate in this study were primarily interested in sharing their experiences. As an example, one migrant (*19P, female, 31 y.o., non-volunteering*) explained that she was not interested in the monetary incentive for their interview participation but “*wanted to help with [this] meaningful research topic*”. The integration of findings related to employment satisfaction by migrants in Japan suggested economic fulfillment to some extent; however, the sample may skew toward privileged migrants.

Even with economic satisfaction, those who legally qualify to reside in modernized Tokyo still find settlement in Japan to be challenging as other policy research has demonstrated [36]. Skilled workers are actively being recruited by the government, which continues to facilitate many practical support services available to foreign residents, from childhood education support [37] and medical interpretation [38] to streamlined permanent residency [39]. On the other hand, a comparative study of highly skilled workers in Sweden and Japan found barriers to migration satisfaction around language barriers and social support [40]. In light of the present findings, it is clear there is more to migrant satisfaction than the benefits of a modernized society or the challenges of language barriers in the workplace.

The final integrative finding was that deep social connection beyond residence was an important factor for mental well-being. Social connectedness is one measure of settlement according to the Australian Settlement Council [17]. Besides providing opportunities to new-comers, putting down social roots can impact satisfaction with a new living environment [41]. The results of mixed methods integration suggest that social factors, above and beyond factors of convenience or economy, are limiting the mental well-being of foreign residents in Japan. Improvement in the bridging social capital of migrants (links of trust and reciprocation between dissimilar groups [42]) in Japan may be have a role to play for improved social cohesion in society at large.

Regression analysis suggested ‘feeling isolation’ to be a cofounding factor in the relationship between volunteering and mental well-being in addition to those factors captured in the joint displays. ‘Not feeling isolated’ is similar to social connection, which has been shown to improve mental health [34], but merely a lack of social connections does not necessarily equate to the extreme isolation the migrants can often experience. These complementary sides of social capital have a complex impact on migrants [6, 42]. Even Japanese residing in Tokyo, also rank in the bottom 25 and 36% of self-rated life satisfaction and community, respectively, according to the OECD’s Better Life Index [43]. Such a challenging social environment may severely affect the mental health of resident migrants.

There are indications that mutually beneficial outcomes are possible with evidence from Germany suggesting that native citizens regard immigrants who engage in community work higher than those with high educational attainment [44]. As it becomes clear that Japan will need international migrants more than the reverse, questions remain about whether Japanese social leaders are willing to facilitate civic and social engagement together with migrants to promote positive mental well-being.

### Strengths and limitations

This study utilized the strengths of both quantitative and qualitative approaches to assess the mental well-being of international migrants by volunteering status outside of a Western context. By integrating results as part of a convergent mixed methods design, more nuanced interpretations about the subjective outcome of mental well-being and associated factors could be made. The partnership with local community NPOs also strengthened the real-world applicability of these findings for future civic engagement by the international migrant community. Any differences in mental well-being between types of volunteer activities (i.e. outreach vs food preparation) were expected to be small. However, non-response bias may have skewed the results towards positive mental well-being scores and quotations because migrants with low mental well-being were perhaps less likely to engage with this study. Differences between the demographics of the quantitative and qualitative participants were mitigated as much as possible with screening but still occurred.

The foreign resident community of Japanese is ethnically heterogeneous. Differences in cultural backgrounds among migrants could have led to bias in the form of different interpretations of survey and interview questions. Additionally, the thinking of some students may be affected by a limited time horizon in Japan. Non-volunteering international migrants who did not have access to online social networking sites may not have been sampled, unlike volunteers who were recruited face-to-face. Results may also be limited by a relatively privileged population of English-speaking skilled foreign workers and students in Japan. Finally, pre-migratory factors which could have predisposed migrants to a certain level of mental well-being were beyond the scope of this study. These limitations were minimized with clear screening questions and explanations but were ultimately inherent in convenience sampling.

## Conclusions

Volunteering status itself was not significantly associated with mental well-being score among international migrants in Japan after adjusting for potential confounding variables. Beyond volunteering, having deeper social connections with the Japanese community is a key to promoting migrant mental well-being. In emerging migration destinations, like Japan, social policy reforms should normalize the equitable participation of minorities in their local community to retain satisfied international migrants who will contribute to the host society. Further research on migrants’ mental well-being and social connectedness should be considered as part of progress towards inclusive societies.

## Supplementary information

**Additional file 1.** Joint display of mental well-being of migrants by volunteering status.

**Additional file 2.** Joint display of mental well-being and employment satisfaction.

## Data Availability

The datasets analyzed in the current study are available from the corresponding author upon reasonable request.

## Abbreviations

COREQ: Consolidated Criteria for Reporting Qualitative Research
IPLII: Immigration Policy Lab Integration Index
MMAT: Mixed Methods Appraisal Tool
NPO: Non-Profit Organization
OECD: Organization for Economic Co-operation and Development
STROBE: Strengthening the Reporting of Observational studies in Epidemiology
UK: United Kingdom
US: United States
WEMWBS: Warwick-Edinburgh Mental Well-Being Scale
