# Supplementary figures and images for: Seeking connection: a mixed methods study of mental well-being and community volunteerism among international migrants in Japan
Source: BMC Public Health. 2020 Aug 20;20:1272. doi: 10.1186/s12889-020-09381-2 (PMC7441705; doi:10.1186/s12889-020-09381-2)

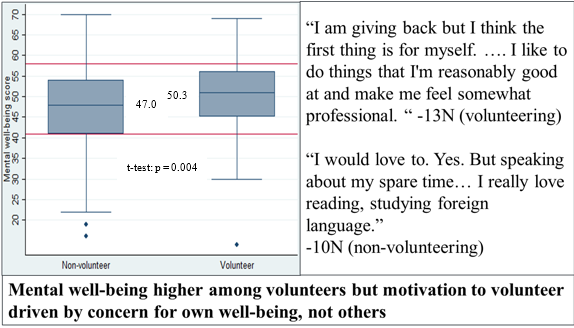

Supplement: Supplementary file 1 — Additional file 1. Joint display of mental well-being of migrants by volunteering status. [file 12889_2020_9381_MOESM1_ESM.docx]

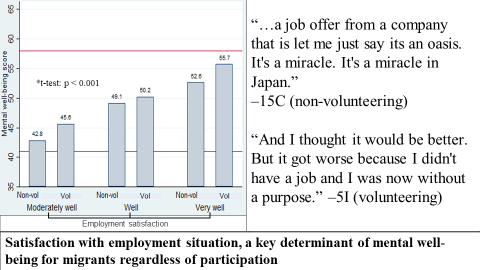

Supplement: Supplementary file 2 — Additional file 2. Joint display of mental well-being and employment satisfaction. [file 12889_2020_9381_MOESM2_ESM.docx]
